# Inflammatory, metabolic, and vascular pathways linking cardiorespiratory fitness to cognition: Results from the IGNITE study

**DOI:** 10.1016/j.bbih.2026.101291

**Published:** 2026-06-25

**Authors:** Patricio Solis-Urra, Kelsey R. Sewell, Audrey M. Collins, Chaeryon Kang, Haiqing Huang, George Grove, Lu Wan, Amani M. Norling, Arthur F. Kramer, Edward McAuley, Jeffrey M. Burns, Charles H. Hillman, Eric D. Vidoni, Anna L. Marsland, M. Ilyas Kamboh, Amanda Szabo-Reed, Renee J. Rogers, Daniel E. Forman, Sandra A. Billinger, John M. Jakicic, Kirk I. Erickson, Lauren E. Oberlin

**Affiliations:** a AdventHealth Research Institute, Neuroscience Institute, Orlando, FL, USA; b Faculty of Education and Social Sciences, Universidad Andres Bello, Viña del Mar, 2531015, Chile; c Centre for Healthy Ageing, Health Futures Institute, Murdoch University, Murdoch, Western Australia, 6150, Australia; d Department of Psychiatry, University of Pittsburgh, Pittsburgh, PA, 15213, USA; e Department of Biostatistics and Health Data Science, School of Public Health, University of Pittsburgh, Pittsburgh, PA, 15261, USA; f Department of Psychology, University of Pittsburgh, Pittsburgh, PA, 15213, USA; g Department of Medicine, Harvard Medical School, Boston, MA, USA; h Beckman Institute for Advanced Science and Technology, University of Illinois at Urbana Champaign, IL, 61801, USA; i Department of Health and Kinesiology, University of Illinois at Urbana-Champaign, Urbana, IL, 61801, USA; j Alzheimer's Disease Research Center, University of Kansas Medical Center, Kansas City, KS, 66160, USA; k Department of Neurology, University of Kansas Medical Center, Kansas City, KS, USA; l Department of Psychology, Northeastern University, Boston, MA, 02115, USA; m Institute for Cognitive & Brain Health, Northeastern University, Boston, MA, USA; n Department of Physical Therapy, Movement, and Rehabilitation Sciences, Northeastern University, Boston, MA, 02115, USA; o Department of Human Genetics, University of Pittsburgh, Pittsburgh, PA, 15261, USA; p Department of Internal Medicine, Division of Physical Activity and Weight Management, University of Kansas Medical Center, Kansas City, KS, 66160, USA; q Department of Medicine (Cardiology and Geriatrics), University of Pittsburgh, and Pittsburgh Geriatrics, Research, Education and Clinical Care (GRECC), Veterans Affairs Pittsburgh Healthcare System, Pittsburgh, PA, USA; r Department of Psychiatry, Weill Cornell Medicine, New York, NY, 10065, USA

## Abstract

**Objectives:**

This study examined whether peripheral biological pathways including inflammation, insulin resistance, and arterial stiffness, partially explain the link between cardiorespiratory fitness (CRF) and cognitive function in older adults.

**Methods:**

In a cross-sectional sample of cognitively unimpaired older adults (N = 648, 71% female, M age = 69.88 *±* 3.75 years), participants completed a comprehensive cognitive battery assessing executive function (EF)/Attentional control, episodic memory, processing speed, working memory, and visuospatial abilities. CRF was measured using a maximal graded exercise test performed on a motorized treadmill. Peripheral biomarkers included low-grade systemic inflammation (Interleukin-6; IL-6), insulin resistance (Homeostatic Model Assessment for Insulin Resistance; HOMA-IR) and arterial stiffness (carotid-femoral pulse wave velocity; cfPWV). Parallel mediation models, adjusted for age, sex, APOE4 carriage, body fat percentage, study site and years of education, tested whether these biomarkers statistically mediated associations between CRF and cognitive performance.

**Results:**

IL-6 emerged as a consistent significant mediator of the relationship between CRF and EF, episodic memory, visuospatial processing and working memory. HOMA-IR statistically mediated the association between CRF and both EF and processing speed. In contrast, cfPWV did not statistically mediate an association between CRF and performance in any cognitive domain.

**Conclusion:**

These findings suggest that low-grade systemic inflammation broadly mediates the relationship between CRF and cognitive function, while metabolic pathways show more domain-specific associations. Together, these results highlight the need for understanding the plural, yet distinct, biological mechanisms by which higher CRF relates to better cognitive performance, with the goal of identifying potential targets for interventions aimed at preserving cognitive health in older adulthood.

## Highlights

• Peripheral biological pathways underlying the CRF–cognition link were examined in cognitively unimpaired older adults.
• Low-grade inflammation broadly mediated the CRF–cognition association.
• Insulin resistance selectively mediated executive function and processing speed.
• Arterial stiffness showed no mediation effect.

## 1 Introduction

Cardiorespiratory fitness (CRF), a physiological marker of aerobic capacity, is a powerful predictor of longevity and is consistently associated with reduced cognitive decline and lower dementia risk (Livingston et al., 2024; Tari et al., 2025). Although higher CRF is linked to numerous central nervous system pathways that support cognitive health, including enhanced synaptogenesis, angiogenesis, and an increase in neurotrophic factor expression (Tari et al., 2025), much remains unknown about the mechanisms underlying these associations. Higher CRF positively influences multiple peripheral pathways including inflammation, metabolic regulation, and vascular function that may support these neuroprotective processes and promote cognitive health (Erickson et al., 2022; Tari et al., 2025). Yet, the precise peripheral mechanisms linking CRF to preserved cognitive health in older adulthood remain unclear.

Several key pathways may underlie the cognitive benefits of higher CRF in older adulthood. Chronic low-grade inflammation, indexed by elevated levels of circulating inflammatory markers such as interleukin-6 (IL-6), predicts increased risk of all-cause dementia (Hallab et al., 2025; Zhao et al., 2024) and greater age-related cognitive decline across multiple domains, including global cognition, memory (Hallab et al., 2025; Zhao et al., 2024), visuospatial processing (Dyer et al., 2024), executive function (EF) and processing speed (Tegeler et al., 2016). Peripheral insulin resistance, measured by the Homeostatic Model Assessment of Insulin Resistance (HOMA-IR), has been associated with increased risk of Alzheimer's disease (AD) (Gutierrez-Tordera et al., 2025) and domain-specific declines in EF and processing speed, but not memory, in older adults without dementia (Lorenzo et al., 2025). Vascular health, including arterial stiffness measured by carotid-femoral pulse wave velocity (cfPWV), has also been related to late life cognitive function (Arvanitakis et al., 2024; Fanelli et al., 2022; Li et al., 2017; Pase et al., 2012), with some evidence of domain- or sex-specific patterns (e.g., stronger links with EF in males) (Sabra et al., 2021). Notably, CRF has been consistently linked to inflammatory, metabolic, and vascular pathways (Albalak et al., 2022; Mamlouk et al., 2025), each of which carries important implications for brain health (Breidenbach et al., 2025; Erickson et al., 2022; Li et al., 2017; Malin et al., 2022).

Despite these established relationships, few studies have examined whether these peripheral pathways statistically mediate associations between CRF and cognition (Barnes and Corkery, 2018). Moreover, available studies have largely evaluated individual pathways in isolation, limiting understanding of the specific mechanisms that may confer cognitive benefits, and whether these relationships differ across cognitive domains. For instance, IL-6 has been shown to mediate the relationship between CRF and working memory in young adults (Hwang et al., 2017), while insulin resistance independently mediates the association between estimated CRF and EF (McIntyre et al., 2023) and memory performance in midlife (Tarumi et al., 2013). Research on arterial stiffness is sparse, though higher CRF and lower arterial stiffness have been jointly associated with better spatial working memory (Kennedy et al., 2018) and EF in older adults (Gauthier et al., 2015). Overall, studies examining these pathways in parallel using a robust sample of older adults with comprehensive evaluations of cognitive performance, CRF, and peripheral biomarkers are scarce.

Clarifying the independent contribution of peripheral mechanisms through which CRF relates to cognition is important for advancing strategies to promote brain health and optimizing exercise-based approaches to support cognitive function. In the current sample, we previously observed that higher CRF was broadly associated with better performance across multiple cognitive domains (Oberlin et al., 2025), yet the pathways driving these relationships remain uncertain. Thus, the aim of this study was to assess whether IL-6, HOMA-IR and cfPWV mediate the association between CRF and cognitive domains susceptible to age-related decline. By testing these pathways simultaneously, we aimed to determine the unique and relative contributions of inflammatory, metabolic, and vascular mechanisms. We hypothesized that independent pathways would differentially mediate CRF and cognition relationships across cognitive domains.

## 2 Methods

### 2.1 Study population and design

Participants were community-dwelling older adults aged 65-80 enrolled in the *Investigating Gains in Neurocognition in an Intervention Trial of Exercise (IGNITE)* study. IGNITE was a multi-site randomized clinical dose-response aerobic exercise trial conducted in Pittsburgh, Kansas City, and Boston (ClinicalTrials.gov registration: NCT02875301). Data collection was harmonized across IGNITE sites using standardized protocols, trained and certified study staff, common assessment manuals, and centralized quality-control procedures. IGNITE followed a manualized set of procedures with annual recertifications to ensure consistency and rigor across sites. Prior to data collection, study staff were required to successfully complete a certification session conducted by the coordinating center staff to ensure harmonization across sites. Additional details regarding multisite standardization procedures are described in the study protocol manuscript (Erickson et al., 2019). The current analysis used only baseline data prior to randomization. Eligible participants had no history or current diagnosis of neurological disorders (e.g., Parkinson's disease, stroke, dementia), major depression, or severe mental illness (e.g., schizophrenia). They also self-reported engaging in less than 60 min of structured moderate-to-vigorous intensity physical activity per week over the prior 6 months. Additional exclusion criteria included active treatment for certain cancers; Type 1 diabetes or uncontrolled or insulin-dependent Type 2 diabetes; and recent cardiovascular events. These events included current treatment for congestive heart failure, angina, uncontrolled arrhythmia, deep vein thrombosis, or other cardiovascular conditions (e.g., heart failure, uncontrolled arrhythmia), as well as myocardial infarction, coronary artery bypass grafting, angioplasty, or other cardiac conditions within the past year. Consensus adjudication was reached by a team of neuropsychologists following a comprehensive cognitive assessment to exclude individuals with probable mild cognitive impairment or dementia. Further details and exclusion criteria can be found in Erickson et al., (2019).

Baseline data were collected between September 2017 and December 2020. All participants provided written informed consent. Ethical approval was obtained by the Institutional Review Board at each participating site: University of Pittsburgh (STUDY19110244), University of Kansas Medical Center (STUDY00140896), and Northeastern University (17-05-02). The present analytic sample included IGNITE baseline participants with available CRF, peripheral biomarker, cognitive, and covariate data. Although this sample overlaps with prior IGNITE reports, the present analysis addresses distinct hypotheses focused on peripheral biological markers linking CRF and cognitive performance.

### 2.2 Cardiorespiratory fitness assessment

CRF was assessed using a graded exercise test performed on a motorized treadmill. Each participant's walking speed was individualized to a pace between 1.5 and 3.5 mph to elicit approximately 70% of their age-predicted maximum heart rate (±5 beats), or rating perceived of exertion (RPE) 11 on the Borg rating scale for participants who reported using beta-blocker medications. As detailed elsewhere (Oberlin et al., 2025), a modified Balke protocol was used (Balke and Ware, 1959), with the speed kept constant while the grade increased by 2% every 2 min. A metabolic cart (Parvo Medics TrueOne 2400; COSMED Quark CPET) with 15 s averaging, along with ECG, blood pressure, and ratings of perceived exertion (RPE) were used throughout the test. Calibration was performed before each test, and manufacturer-recommended quality control procedures were completed every six months. CRF was assessed as peak oxygen consumption (VO_2_) before volitional exhaustion or symptom-limited termination. Assessors also recorded whether the test met standard ACSM criteria(Medicine ACoS, 2020) for defining maximal effort on a graded exercise test, including: a VO_2_ plateau (<0.15 L/min or <2.0 mL/kg/min increase between workloads), RER ≥1.10, heart rate within 10 beats of the age-predicted maximum, or an RPE ≥17. All exercise tests were administered by certified staff following standards established by the American College of Sports Medicine (ACSM). Participants who met any ACSM-defined absolute or relative contraindications for graded exercise testing were not permitted to continue (Medicine ACoS, 2020).

### 2.3 Assessment of peripheral pathways

Assessment procedures for peripheral blood biomarkers have been described previously (Jain et al., 2025; Olvera-Rojas et al., 2025). Briefly, venous blood was collected between 7:00 a.m. and 1:00 p.m. after an overnight fast and at least 12 h of abstinence from exercise, alcohol, and headache medication, as well as abstinence from nicotine for 1 h prior to the session. Anyone non-compliant with these instructions was rescheduled for the blood draw. Concentrations of IL-6 were quantified in plasma using Simple Plex assays and the antibody-based Ella™ system (ProteinSimple, Biotechne), following the manufacturer's protocols, and expressed as pg/mL. Fasting insulin was measured using a sandwich immunoassay with electrochemiluminescence detection, and glucose was quantified using the hexokinase G-6-PDH method (Beckman Coulter). These values were used to calculate HOMA-IR according to the following equation: fasting insulin (μU/mL) × fasting glucose (mg/dL)/405. Insulin and glucose samples were run in duplicate, while IL-6 samples were run in triplicate, with coefficients of variation below 10%. Supine cfPWV was measured using applanation tonometry (SphygmoCor XCEL) using standard protocols after 10 min of supine rest (Norling et al., 2025). Two cfPWV measurements were collected for each participant. If either measurement failed quality standards or if the two values differed by more than 0.5 m/s, a third measurement was taken. The final cfPWV value was calculated as the average of all trials that met quality assurance criteria (Norling et al., 2025).

### 2.4 Cognitive outcomes

Cognition was assessed using a comprehensive neuropsychological battery of paper-and-pencil and computerized assessments. The battery was delivered across two days by annually certified psychometricians. The cognitive assessment included measures of *processing speed* (Letter Comparison Test, Digit Symbol Substitution Test, Trail Making Test, Part A), *episodic memory* (Brief Visuospatial Memory Test, Picture Sequencing Test, Hopkins Verbal Learning Test, Logical Memory Task, Montreal Cognitive Assessment (MoCA) free recall, Verbal Paired Associates), *working memory* (N-Back Working Memory Task, Spatial Working Memory Task, List Sorting Working Memory Task), *visuospatial abilities* (Matrix Reasoning, Spatial Relations, MoCA Clock Draw), and *executive function (EF)/attentional control* (Flanker Task, Stroop Task, Dimensional Change Card Sort task, Trail Making Test, Part B). Five previously established latent factors were used to measure performance in each domain (Oberlin et al., 2025), with higher values indicating better performance.

### 2.5 Covariates and moderators

Covariates included age based on self-reported date-of-birth, sex (defined as sex assigned at birth), apolipoprotein (APOE) 4 allele carrier status, total body fat %, study site, and years of education. APOE genotyping was performed using TaqMan assays to detect two APOE single-nucleotide polymorphisms (rs429358 and rs7412). Individuals with at least one *APOE4* allele (i.e., 2/4, 3/4, or 4/4 genotypes) were classified as *APOE4* carriers. Because adiposity is associated with peripheral pathways of interest (Herzog et al., 2025; James et al., 2021; Le Thuc and Garcia-Caceres, 2024), total body fat percentage (%), quantified from whole body dual energy x-ray absorptiometry (DXA) scans (GE Lunar iDXA; Madison, WI, USA), was included as a covariate. Total body fat % was produced by the enCORE (version 18) manufacturer software following a total body DXA scan. Participants were scanned in a standard supine position with head at the top of the scanning zone, arms at sides (palms inward) maintaining an air gap from the trunk, and legs extended with ankles secured by Velcro. MirrorImage processing was applied when positioning exceeded the scan field, prioritizing alignment of the right side. Type 2 diabetes was identified through self-reported health history and/or current use of type 2 diabetes medication. Body mass index (BMI) was calculated as weight in kilograms divided by height in meters squared, using measurements obtained from calibrated stadiometers and scales while wearing light clothing and no shoes.

### 2.6 Statistical analysis

Analyses were conducted using R version 4.5.0 (R Foundation for Statistical Computing, Vienna, Austria, RRID:SCR_0019054). Descriptive statistics are presented as mean ± standard deviation, or number (percentage), as appropriate. IL-6 and HOMA-IR values were log-transformed prior to analysis to reduce skewness. cfPWV was retained on its original scale because its distribution was acceptable for modeling and because the original units are clinically interpretable. Parallel mediation analyses were performed to test whether IL-6, HOMA-IR or cfPWV statistically mediated associations between CRF (predictor) and cognitive performance (outcomes). Parallel mediation analysis estimates the indirect effect of the predictor on the outcome through multiple mediators simultaneously, where the overall indirect effect is calculated by summing the indirect effects from each mediator. In parallel mediation, the mediators are assumed to act independently, enabling examination of distinct biological pathways linking CRF to cognition. Five parallel mediation models were performed, one for each cognitive domain (EF/attentional, episodic memory, processing speed, visuospatial processing, working memory), using the *lavaan* package in R. All models adjusted for age, sex, *APOE4* carriage, total body fat %, study site, and years of education. Statistical mediation was examined using bias–corrected accelerated (BCa) bootstrap confidence intervals (CIs) for the indirect effect, based on 5000 bootstrapped resamples. Indirect effects were considered significant if the 95% CIs did not contain zero. The proportion mediated was calculated as the ratio of the indirect effect to the total effect (Mackinnon et al., 2004). For sensitivity analyses, total body fat % was substituted for BMI. Additional sensitivity analyses were performed excluding participants with diabetes.

## 3 Results

Table 1 shows the descriptive characteristics of the sample. The IGNITE sample included 648 older adults with a mean age of 69.88 ± 3.75 years, 71% women, with an average of 16.32 ± 2.21 years of education. Pearson correlations between the peripheral pathways are 0.3 for IL-6 and HOMA-IR, 0.07 for IL-6 and cfPWV, and 0.14 for HOMA-IR and cfPWV.

**Table 1:** Descriptive characteristics of IGNITE sample.

Characteristic	N	N = 648
Age, yr,	648	69.88 ± 3.75
Education, yr,	648	16.32 ± 2.21
Sex, n %, n (%)	648	
Female		461 (71%)
Male		187 (29%)
Site, n (%)	648	
Kansas		214 (33%)
Northeastern		215 (33%)
Pittsburgh		219 (34%)
*APOE4* carrier, n (%)	640	174 (27%)
Total body fat, %,	647	40.15 ± 7.49
Body-mass index, kg/m^2^,	648	29.77 ± 5.75
VO_2peak,_ (mL/kg/min),	648	21.68 ± 5.06
Interleukin-6, pg/ml,	615	0.91 ± 0.64
HOMA-IR,	595	0.78 ± 0.87
cfPWV, m/s,	610	9.05 ± 2.03
Diabetes status, n (%)	648	
No		545 (84%)
Yes		103 (16%)
EF/attentional control,	648	0.00 ± 0.59
Processing speed,	648	0.00 ± 0.62
Working memory,	648	0.00 ± 0.54
Episodic memory,	648	0.00 ± 0.61
Visuospatial processing,	648	0.00 ± 0.56

Note: Unless otherwise specified, data are presented as mean ± standard deviation. Abbreviations: *APOE4*, apolipoprotein E4 variant. EF, Executive function. HOMA-IR, Homeostasis Model of Insulin Resistance. cfPWV, carotid-femoral pulse wave velocity.

### 3.1 Cardiorespiratory fitness associations with peripheral pathways

**Fig. 1:**
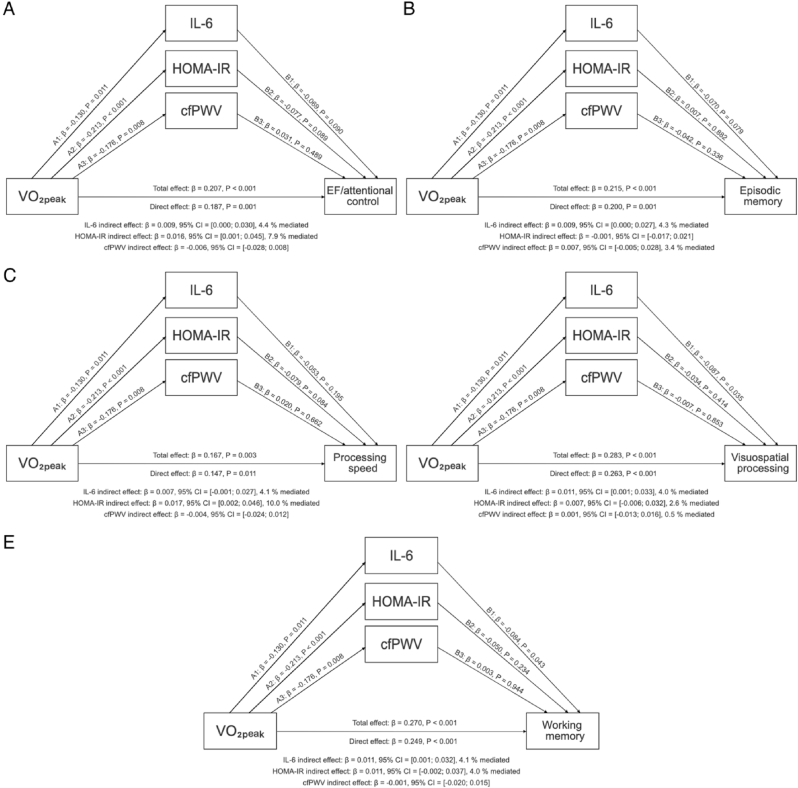
Results of parallel bootstrapped mediation analyses examining the mediating roles of interleukin-6 (IL-6), the Homeostasis Model of Insulin Resistance (HOMA-IR), and carotid-femoral pulse wave velocity (cfPWV) on cognition. All mediation analyses used 5000 resamples and bias-corrected accelerated (BCa) confidence intervals. Covariates included age, education, sex, site, *APOE4* genotype, and total body fat percentage (n = 544).

As illustrated in Fig. 1 (path A), higher CRF was associated with lower IL-6 (A1: β = −0.130, P = 0.011), lower HOMA-IR (A2: β = −0.213, P < 0.001), and lower cfPWV (A3: β = −0.176, P = 0.008).

### 3.2 Parallel mediation results

Fig. 1 displays the total, direct, and indirect effects across the five cognitive domains. As previously reported, higher CRF was significantly associated with better performance across all cognitive domains (Oberlin et al., 2025). In the domain of EF/attentional control (Fig. 1A), both IL-6 and HOMA-IR independently mediated the association between CRF and performance. Specifically, IL-6 accounted for 4.4% of the association (indirect effect (IE): β = 0.009, 95% CI: = 0.000 to 0.030) and HOMA-IR accounted for 7.9% (IE: β = 0.016, 95% CI: = 0.001 to 0.045) of the association. IL-6 mediated 4.3% (IE: β = 0.009, 95% CI: = 0.000 to 0.027) (Fig. 1B) of the association with episodic memory, 4.0% (IE: β = 0.011, 95% CI: = 0.001 to 0.033) (Fig. 1D) of the association with visuospatial processing, and 4.1% (IE: β = 0.011, 95% CI: = 0.001 to 0.032) of the association with working memory (Fig. 1E). HOMA-IR mediated 10.0% (IE: β = 0.017, 95% CI: = 0.002 to 0.046) of the association between CRF and processing speed (Fig. 1C). cfPWV did not independently mediate the relationship between CRF and performance in any cognitive domain (β range = −0.006 to 0.007, 95% CI range: −0.024 to 0.028). Sensitivity analyses replacing total fat % with BMI and excluding participants with type 2 diabetes yielded generally consistent results (Supplementary Figs. 1 and 2), though the indirect association of the HOMA-IR path with processing speed and EF/attentional control was trending in the same direction but was no longer significant in the non-diabetic subsample.

## 4 Discussion

The aim of this study was to examine whether IL-6, HOMA-IR, and cfPWV statistically mediate the association between CRF and cognitive performance in cognitively unimpaired older adults. We observed that the relationship between CRF and cognition showed broad indirect associations through IL-6 across most cognitive domains. HOMA-IR exhibited selective indirect associations in the domains of EF/attentional control and processing speed, while cfPWV did not show statistically significant indirect associations across any cognitive domains.

Higher CRF was favorably associated with each peripheral pathway (IL-6, HOMA-IR, and cfPWV), consistent with prior reports (Albalak et al., 2022; Breidenbach et al., 2025; Erickson et al., 2022; Kennedy et al., 2018; Mamlouk et al., 2025; Tarumi et al., 2013). When examining these pathways simultaneously in parallel mediation models, we observed selective indirect associations of specific pathways and cognitive domains. Specifically, IL-6 partially mediated the associations of CRF with EF/attentional control, episodic memory, visuospatial memory and working memory, suggesting that circulating inflammation may be involved in the association between CRF and multi-domain cognitive performance. By simultaneously evaluating inflammatory, metabolic, and vascular pathways, the present findings suggest that immune-related processes may contribute to the relationship between fitness and cognition. However, because IL-6 was the only inflammatory marker examined, these findings should be interpreted as supporting the relevance of immune processes rather than a specific immune mechanism. While the observed indirect effects were small, such effects may still be meaningful at the population level, particularly for complex phenotypes such as cognition. Furthermore, given the broad and multifaceted physiological influences of exercise and fitness, modest indirect effects through any single pathway would be expected. Elevated IL-6 levels reflect chronic, low-grade inflammation, which has been associated with maladaptive processes in the brain, including disruptions of blood-brain barrier integrity, sustained astrocyte and microglial activation, and synaptic dysfunction (Le Thuc and Garcia-Caceres, 2024). These processes are also influenced by CRF, suggesting a biologically plausible statistical pathway linking higher CRF with lower inflammatory signaling and better multi-domain cognitive performance (Greene et al., 2024; Lyra et al., 2021). Importantly, these effects appear to be independent of insulin resistance or arterial stiffness, emphasizing systemic inflammation as a distinct pathway linking CRF and cognitive function. Future studies should test whether maintaining higher aerobic fitness reduces chronic inflammation and supports cognitive health in older adults. However, establishing causal influence requires experimental manipulations to determine whether interventions that improve CRF directly reduce IL-6 and subsequently enhance cognitive performance.

HOMA-IR selectively mediated the association of CRF with EF/attentional control and processing speed, while its influence on episodic memory and other domains was minimal and non-significant. This pattern suggests that distinct physiological processes related to CRF may differentially support specific cognitive processes in older adulthood. Insulin-resistance has been linked to detrimental brain health decades before clinical symptoms of AD and related dementias appear (Le Thuc and Garcia-Caceres, 2024), including white matter abnormalities and disrupted resting-state functional networks (Cui et al., 2022), which may primarily impact EF and processing speed abilities (Cui et al., 2022). In contrast, episodic memory and visuospatial domains, linked to hippocampal and medial temporal lobe integrity (Cui et al., 2022), may remain relatively preserved in the context of mild insulin resistance(Arvanitakis et al., 2010; Biswas et al., 2025). Interestingly, and in line with the current results, prior studies have shown that neither working memory, episodic memory, nor visuospatial processing are associated with diabetes status (Arvanitakis et al., 2010; Biswas et al., 2025). These cross-sectional findings suggest that HOMA-IR may be a relevant metabolic correlate of EF and processing speed and a candidate pathway for future longitudinal and intervention studies.

The lack of a mediating pattern of cfPWV suggests that arterial stiffness may not be a primary pathway linking CRF to cognitive health independent of inflammatory and metabolic mechanisms. Conversely, these findings may also reflect the relatively narrow vascular profile of this community-dwelling sample (Norling et al., 2025). Although arterial stiffness increases with age, only 25% of participants exceeded age-referenced normative cfPWV thresholds (≥9.7 m/s for ages 60–69; ≥10.6 m/s for ages ≥70 (Reference Values for Arterial Stiffness, 2010), suggesting a relatively lower burden of marked aortic stiffening in this cohort. Early stage cfPWV variability may predominantly reflect large artery rather than peripheral cerebral microvascular function, potentially reducing its direct association with cognition (Le Thuc and Garcia-Caceres, 2024). Supporting this, previous studies have shown that associations between CRF and cognition are not consistently mediated by structural cerebrovascular lesions such as white matter hyperintensities or lacunes in older adults (Freudenberger et al., 2016). Moreover, this sample included adults ages 65-80, and prior evidence suggests that the cfPWV effects on cognition may interact with age, being more pronounced at more advanced ages (Elias et al., 2009). This aligns with meta-analytic findings showing attenuated associations in healthier, lower-risk cohorts (Liu et al., 2021). Expanding future analyses to encompass a wider spectrum of cardiovascular health and disease states may help disentangle these associations and strengthen the validity of our conclusions.

While inflammation can contribute to insulin resistance, metabolic dysregulation, and vascular impairment (James et al., 2021; Sotak et al., 2025; Tilg et al., 2025), the causal sequence between these pathways and cognitive outcomes remains complex and inconclusive. Our study focused on IL-6, HOMA-IR, and cfPWV in parallel, but other peripheral mechanisms may also contribute to the CRF-cognition association (e.g., dyslipidemia). Other possible molecular mediators such as brain-derived neurotrophic factor (BDNF), insulin-like growth factor (IGF-1), structural and functional brain features including cortical and subcortical volumes, and behavioral and psychosocial factors such as mood and sleep, have all been suggested as possible mediators (Stillman et al., 2020; Tari et al., 2025). Future studies integrating these additional pathways will be critical for further evaluating the specific mechanisms by which CRF is associated with better cognitive health in late adulthood (Tinney et al., 2025).

In summary, this study suggests that higher CRF is associated with cognitive performance through statistical indirect associations involving peripheral markers, with systemic inflammation playing a broad role and insulin resistance relating to specific domains. Clinically, these findings highlight the importance of maintaining or improving aerobic fitness in older adulthood as a modifiable factor associated with cognitive function in older adulthood. Furthermore, identifying domain-specific mediators offers a framework for targeted CRF-enhancing interventions aimed at reducing dementia risk and optimizing brain health across aging populations.

This study has several notable strengths such as rigorous population phenotyping, robust measurement protocols, and a well-defined conceptual framework for measuring cognition. CRF and arterial stiffness were assessed using gold-standard methods, specifically graded exercise testing and cfPWV. Our analyses incorporated multiple peripheral pathways, allowing for a comprehensive evaluation of potential mechanisms linking CRF and cognitive performance. Cognitive function was measured using an extensive battery of standardized tests and latent factors representing core cognitive domains that were empirically derived using a confirmatory factor analysis (Oberlin et al., 2025), thereby strengthening internal validity. Additionally, accounting for body fat percentage reduced potential confounding by adiposity. However, the cross-sectional design precludes causal inference and the ability to determine the directionality of associations. Thus, mediation models are interpreted as tests of hypothesized statistical pathways rather than evidence of causal mediation or temporal ordering. Because inference for the indirect effects was based primarily on bootstrap confidence intervals rather than p-values, we did not apply a formal false discovery rate correction in the main analysis. However, the mediation results should be interpreted cautiously, especially the borderline findings, because these may be sensitive to correction for multiple testing. Longitudinal studies and experimental manipulation of CRF and body composition will help to disentangle the trajectories of these relationships, including how changes in body composition may impact peripheral mediators and, in turn, cognitive health. Our sensitivity analysis revealed that excluding participants with type 2 diabetes minimally altered IL-6 associations, while indirect associations of HOMA-IR with processing speed and EF/attention control were directionally similar and trending but non-significant. These findings indicate that presence of diabetes may influence the strength of associations with HOMA-IR. Future studies with a larger proportion of participants with diabetes are needed to determine whether HOMA-IR represents a particularly relevant pathway linking CRF to cognition in those with diabetes. Although analyses adjusted for key demographic, genetic, adiposity, and site-related covariates, residual confounding from medication use, comorbidities, diet, sleep, and other lifestyle factors cannot be excluded. However, main findings remained consistent in sensitivity analyses additionally adjusting for diabetes status and overall medical comorbidity burden (not shown). Additionally, because blood was collected between 7:00 a.m. and 1:00 p.m., circadian variation in IL-6 and individual differences in sleep-wake timing may have influenced inflammatory measurements, likely increasing variability and attenuating observed associations. Additionally, participants were recruited as part of a clinical trial and met specific inclusion criteria, including low levels of physical activity, which may have restricted the range of CRF in the sample. Nevertheless, our results suggest that even modest variations in CRF is linked to physiological benefits that support cognitive health. In addition, the sample had a higher proportion of females and was comprised of cognitively unimpaired older adults that had, on average, greater years of education than national averages. This sample composition may reflect recruitment patterns, eligibility criteria, and willingness to participate in an intensive exercise and cognitive-health study. Future research should aim to replicate these findings in more diverse populations, including a greater proportion of males, other comorbidities, broader age ranges, various levels of education, and individuals with cognitive impairment, to strengthen generalizability and broaden understanding of the mechanisms underlying the CRF-cognition relationship. APOE4 status was included as a covariate because of its relevance to Alzheimer's disease risk and neuroinflammatory pathways; however, APOE4 moderation of the indirect associations was not the primary focus of this analysis and should be examined in future studies with adequate power for genotype-stratified mediation analyses.

In conclusion, our findings suggest that the association between CRF and cognitive performance is broadly linked to systemic inflammation, as reflected by IL-6, and selectively by insulin resistance (HOMA-IR). Future research should explore additional biological mechanisms and test whether targeted interventions that improve CRF modify these pathways and cognitive outcomes over time.

## Declaration of generative AI and AI-assisted technologies in the manuscript preparation process

During the preparation of this work, the author(s) used ChatGPT for minor language refinement. The author(s) reviewed and edited the output as needed and take full responsibility for the content of the published article.

## CRediT authorship contribution statement

**Patricio Solis-Urra:** Conceptualization, Data curation, Formal analysis, Methodology, Visualization, Writing – original draft, Writing – review & editing. **Kelsey R. Sewell:** Writing – review & editing. **Audrey M. Collins:** Writing – review & editing. **Chaeryon Kang:** Methodology, Writing – review & editing. **Haiqing Huang:** Data curation, Writing – review & editing. **George Grove:** Investigation, Project administration, Writing – review & editing. **Lu Wan:** Writing – review & editing. **Amani M. Norling:** Writing – review & editing. **Arthur F. Kramer:** Writing – review & editing. **Edward McAuley:** Writing – review & editing. **Jeffrey M. Burns:** Writing – review & editing. **Charles H. Hillman:** Writing – review & editing. **Eric D. Vidoni:** Writing – review & editing. **Anna L. Marsland:** Writing – review & editing. **M. Ilyas Kamboh:** Writing – review & editing. **Amanda Szabo-Reed:** Writing – review & editing. **Renee J. Rogers:** Writing – review & editing. **Daniel E. Forman:** Writing – review & editing. **Sandra A. Billinger:** Writing – review & editing. **John M. Jakicic:** Writing – review & editing. **Kirk I. Erickson:** Conceptualization, Funding acquisition, Investigation, Methodology, Resources, Supervision, Writing – review & editing. **Lauren E. Oberlin:** Conceptualization, Supervision, Writing – review & editing.

## Declaration of competing interest

The authors declare the following financial interests/personal relationships which may be considered as potential competing interests: John M. Jakicic reports a relationship with Wondr Health, Inc that includes: board membership. Renee J. Rogers reports a relationship with AstraZeneca Pharmaceuticals LP that includes: consulting or advisory. Renee J. Rogers reports a relationship with Neurocrine Biosciences Inc that includes: consulting or advisory. Renee J. Rogers reports a relationship with Wondr Health, Inc. that includes: consulting or advisory. Renee J. Rogers reports a relationship with Seca Ltd that includes: consulting or advisory. If there are other authors, they declare that they have no known competing financial interests or personal relationships that could have appeared to influence the work reported in this paper.
