# Supplementary material for: Inflammatory, metabolic, and vascular pathways linking cardiorespiratory fitness to cognition: Results from the IGNITE study
Source: Brain Behav Immun Health. 2026 Jun 25;55:101291. doi: 10.1016/j.bbih.2026.101291 (PMC13324667; doi:10.1016/j.bbih.2026.101291)
Supplement: Multimedia component 1 [file mmc1.docx]

Supplementary material


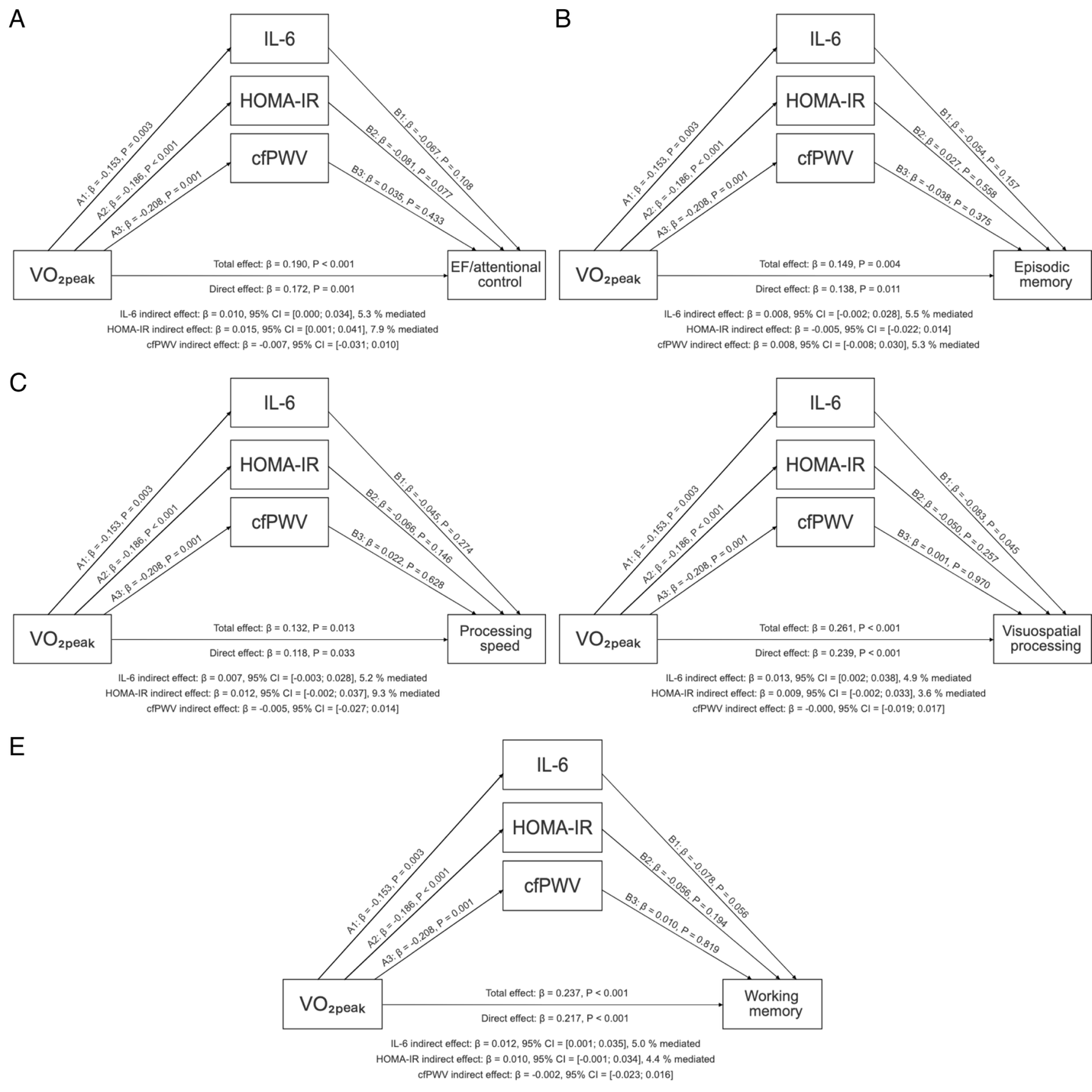


**Figure Sup 1.** Results of parallel bootstrapped mediation analyses examining the mediating roles of interleukin-6 (IL-6), the Homeostasis Model of Insulin Resistance (HOMA-IR), and carotid-femoral pulse wave velocity (cfPWV) on cognition replacing body fat % with BMI as a covariate. All mediation analyses used 5,000 resamples and bias-corrected accelerated (BCa) confidence intervals. Covariates included age, education, sex, site, *APOE4* genotype and body mass index (n = 544).

**
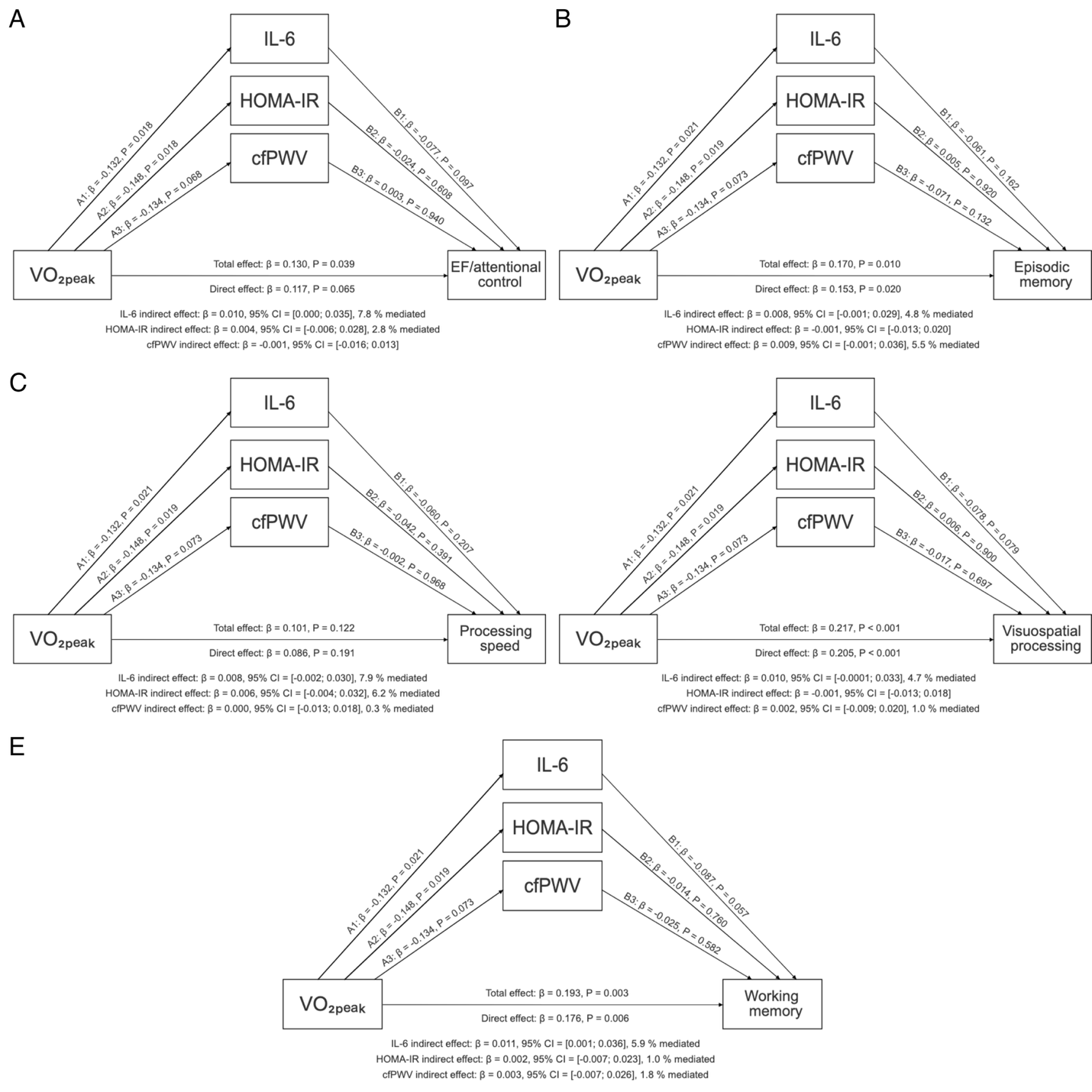
**

**Figure Sup 2.** Results of parallel bootstrapped mediation analyses examining the mediating roles of interleukin-6 (IL-6), the Homeostasis Model of Insulin Resistance (HOMA-IR), and carotid-femoral pulse wave velocity (cfPWV) on cognition. All mediation analyses used 5,000 resamples and bias-corrected accelerated (BCa) confidence intervals. Covariates included age, education, sex, site, *APOE4* genotype and total body fat percentage in people without diabetes (n= 457).
